# Mechanism of action of exercise regulating intestinal microflora to improve spontaneous hypertension in rats

**DOI:** 10.17305/bb.2024.11174

**Published:** 2024-10-30

**Authors:** Yu Li, Xiaoju Song, Lianjing Dai, Yangyi Wang, Qiong Luo, Lei Lei, Yunfei Pu

**Affiliations:** 1 Department of Cardiology, Chongqing General Hospital, Chongqing University, Chongqing, China

**Keywords:** Exercise, intestinal microbial flora, spontaneous hypertension, fecal bacteria transplantation, sympathetic nerve activity

## Abstract

Hypertension is a prevalent cardiovascular disease. Exercise is widely recognized as an effective treatment for hypertension, and it may also influence the composition of the intestinal microflora. However, it remains unclear whether exercise can specifically regulate the intestinal microflora in the context of hypertension treatment. In this study, tail blood pressure in spontaneously hypertensive rats (SHR) was measured using a blood pressure meter after exercise intervention and fecal bacteria transplantation following exercise. Blood lipid levels were assessed using an automatic biochemical analyzer, and 16S rRNA sequencing was employed to analyze the intestinal microflora. Histological examinations of ileal tissue were conducted using HE and Masson staining. Intestinal permeability, inflammatory status, and sympathetic activity were evaluated by measuring the levels of diamine oxidase, D-lactic acid, C-reactive protein, interleukin-6, tumor necrosis factor-α, lipopolysaccharide, norepinephrine, angiotensin II, cyclic adenosine monophosphate, and cyclic guanosine monophosphate. Exercise was found to reduce blood pressure and blood lipid levels in SHR. It also improved the composition of the intestinal microflora, as evidenced by a reduced *Firmicutes/Bacteroidetes* ratio, an increase in bacteria that produce acetic and butyric acid, and higher Chao 1 and Shannon diversity indices. Furthermore, exercise reduced the thickness of the fibrotic and muscular layers in the ileum, increased the goblet cell/villus ratio and villus length, and decreased intestinal permeability, inflammatory markers, and sympathetic nerve activity. The intestinal microbial flora regulated by exercise demonstrated similar effects on hypertension. In conclusion, exercise appears to regulate the intestinal microflora, and this exercise-induced change in flora may contribute to improvements in hypertension in rats.

## Introduction

Hypertension is a chronic disease marked by elevated arterial blood pressure and is a major risk factor for cardiovascular and cerebrovascular diseases [1]. It is also the world’s leading cause of death [2]. Each year, hypertension causes 9.4 million deaths globally, and 212 million people experience health deterioration due to the disease [3]. The number of people with hypertension worldwide doubled from 1990 to 2019, reaching 1.28 billion, which places a heavy burden on families and healthcare systems [4]. Over 90% of hypertension cases are classified as spontaneous, meaning they lack a clear, direct cause [5], but the exact underlying mechanisms remain unclear. Current treatments primarily focus on controlling blood pressure rather than curing the condition [6]. Therefore, the outlook for hypertension remains challenging, and effective treatment is a worldwide problem. It is essential to further explore the pathogenesis of hypertension to identify new therapeutic targets.

In recent years, exercise rehabilitation has gained recognition as a non-drug approach for managing hypertension due to its many benefits [7, 8], and it has become widely used in clinical rehabilitation settings. Research shows that aerobic exercise, resistance training, and a combination of both can significantly reduce blood pressure [9, 10]. Strong evidence supports the benefits of regular physical activity in preventing and managing hypertension [11]. Studies suggest that the blood pressure-lowering effects of exercise are associated with increased nitric oxide (NO) bioavailability in vascular endothelial cells, improved autonomic nervous system regulation, enhanced mitochondrial function, and reduced oxidative stress [12–15]. However, given the complex pathogenesis of hypertension, it is still unclear whether additional mechanisms are involved in the benefits of exercise rehabilitation.

The human gut hosts a diverse microbiota, including bacteria, fungi, archaea, viruses, and protozoa, with bacteria being the most abundant. Advances in sequencing technology have revealed that gut microbes are involved in the physiological and pathological processes of hypertension. When the intestinal mucus barrier, mechanical barrier, and immune barrier are compromised, bacteria and their metabolites can enter the bloodstream, affecting blood pressure and the cardiovascular system [16]. Studies have shown that a high-salt diet inhibits Lactobacillus in the gut, reduces indole products, and increases Th17 cells; supplementation with Lactobacillus can counteract high-salt-induced hypertension [17, 18]. Microbial metabolites in the gut can help regulate blood pressure through mechanisms, such as vasodilation, renal sodium/potassium regulation, immune modulation, and neurotransmitter activity [19]. Given the strong connection between gut microbes and hypertension, the gut microbiome has emerged as a potential target for hypertension treatment.

Exercise has been shown to influence the gut microbiota. Research indicates that athletes tend to have a greater diversity of gut microbial species [20]. Higher levels of physical activity and cardiorespiratory fitness correlate with increased diversity and abundance of specific microbial species, as well as higher concentrations of short-chain fatty acids (SCFAs) [21]. Studies suggest that exercise training can positively alter gut flora, promoting beneficial bacteria and reducing harmful ones, which improves overall gut health and metabolic efficiency [22]. Moderate-intensity exercise has also been shown to enhance exercise performance in mice and influence the metabolic activity of core gut bacteria [23]. Exercise can increase the diversity and abundance of gut microbes, boost SCFA production, and modulate blood pressure. For example, athletes have been found to have relatively higher SCFA levels [24], and exercise can improve the diversity and richness of the gut microbiome, regulate SCFAs and bile acids, and reduce inflammation. This evidence suggests that exercise has a positive impact on gut health.

In summary, while exercise rehabilitation is known to influence gut microbiota, its direct effects on blood pressure regulation have not been fully explored. This study aims to address this gap by using spontaneously hypertensive rats (SHR) and SHR treated with fecal microbiota transplantation following exercise to investigate how exercise might improve spontaneous hypertension. By assessing blood pressure, blood lipids, gut microbiota composition, intestinal pathology, intestinal permeability, inflammation, and sympathetic nerve activity in these rats, this study seeks to provide new insights into hypertension treatment.

## Methods

### Animal grouping and processing

Six normotensive Wistar Kyoto (WKY) rats and 36 SHR, all 8-week-old SPF males weighing between 260 and 310 g, were provided by Charles River Laboratory. The rats were housed under standard conditions (temperature: 20–24 ^∘^C, relative humidity: 50%–70%, light/dark cycle: 12 h/12 h). This experiment was approved by the Animal Ethics Committee. The feed was sterilized by baking at 100 ^∘^C for 1 h before being provided to the animals.

The six WKY rats were used as the blank control group (WKY). The SHR rats were randomly divided into two groups: a hypertension control group (SHR-S) and an exercise intervention group (SHR-E), with six rats in each group. Neither the WKY group nor the SHR-S group received any treatment. The SHR-E group underwent an 8-week exercise intervention program that combined resistance and aerobic exercises, six days per week.

The exercise regimen for the SHR-E group was as follows.

*Resistance Exercise:* Conducted on Mondays, Wednesdays, and Fridays each week. A self-made ladder with an 85∘ incline and a 1-meter length was used for tail-weight-bearing climbing exercises. Balloons with an initial mass of 1 g were attached to the rats’ tails. During weeks 1–5, and 8 of the intervention, water was added to the balloons to increase the weight to 10%, 20%, 30%, 50%, and 70% of each rat’s body weight, respectively. Each rat climbed the ladder three times per session, with a total of two sessions per day and a 2-minute rest interval between sessions. The ladder had a 1 cm step spacing.

*Aerobic Exercise:* Conducted on Tuesdays, Thursdays, and Saturdays using a KW-PT rat treadmill for non-weight-bearing exercise. In the first week of the intervention, the treadmill speed was set to 10–15 m/min, with a duration of 30 min per day and a slope of 0%. From the second to the eighth week, the treadmill speed was maintained at 15 m/min, with an increased duration of 60 min per day, and the slope remained at 0%.

### Fecal bacteria transplantation

Using a manual rough filtration and centrifugal enrichment method, 10 g of fresh feces from WKY, SHR-S, and SHR-E rats were weighed into a sterile beaker. Then, 50 mL of sterile 0.9% NaCl solution was added, and the mixture was stirred thoroughly. The mixture was filtered through 2, 4, and 8 layers of sterile medical gauze. The filtered samples were centrifuged at 10,000 r/min for 10 min, and the resulting pellet was re-suspended in 10 mL of sterile 0.9% NaCl solution, yielding a colorless and odorless fecal bacteria solution. This solution was stored in a freezer at --80 ^∘^C [25].

The SHR rats were randomly divided into four groups: PBS buffer group (S-PBS), WKY fecal transplantation group (S-WKY), SHR-S fecal transplantation group (S-SHR-S), and SHR-E fecal transplantation group (S-SHR-E), with six rats in each group. The S-WKY, S-SHR-S, and S-SHR-E groups received the corresponding fecal bacteria solution by enema once a day for eight weeks, while the S-PBS group received an equivalent volume of PBS only.

### Blood pressure and HR measurement

At room temperature, in a quiet and awake state, the systolic blood pressure (SBP), diastolic blood pressure (DBP), mean arterial pressure (MAP), and HR of the tail artery in each group of rats were measured using a non-invasive blood pressure monitor (BP-300A, Chengdu Taimeng, China). After the blood flow waveform stabilized, data were recorded, with the highest and lowest values excluded, and the average value calculated.

### Blood lipid index detection

At the end of the experiment, blood was collected from the orbital vein of each rat, centrifuged at 4 ^∘^C and 3000 r/min for 10 min, and the serum was collected. The serum levels of total cholesterol (TC), triglycerides (TG), and low-density lipoprotein (LDL) were measured using an automatic biochemical analyzer (Mindray BS-350S, Nanjing Beideng Medical, China).

### 16S rRNA sequencing

Rat feces samples were collected and stored in liquid nitrogen. DNA was extracted using the E.Z.N.A.™ Mag-Bind Soil DNA Kit. PCR amplification targeted the V3-V4 hypervariable region of the 16S rRNA gene, using universal primers. The amplified DNA was then purified with Hieff NGS™ DNA Selection Beads (Yeasen, 10105ES03, China). To construct the sequencing library, Illumina-compatible bridge PCR primers were used for a second round of amplification. Library size was verified by 2% agarose gel electrophoresis, and concentration was measured with a Qubit 3.0 fluorometer. Sequencing was performed on the Illumina MiSeq platform (Illumina, USA) by Bio-Engineering Co., Ltd. (Shanghai, China).

The sequencing data were processed with PEAR (v0.9.8) to merge paired-end reads. Operational taxonomic units (OTUs) were clustered using Usearch software (11.0.667), with sequences sharing ≥97% similarity grouped into the same OTUs. Taxonomic classification was performed with the Mothur algorithm, and alpha diversity indices, including Chao1 richness and Shannon diversity, were calculated using R software (v3.6.0).

### Pathological staining

Rat ileum tissue samples were fixed with 4% paraformaldehyde, dehydrated in a graded ethanol series, embedded in paraffin, and then sectioned into 4-µm-thick slices. The sections were stained with hematoxylin–eosin (HE) and Masson’s trichrome.

*HE staining:* Hematoxylin (H8070, Solarbio, Beijing, China) was applied for 8 min, followed by differentiation with 1% acidic alcohol for 2 s, anti-blue treatment with tap water for 3 min, and counterstaining with 0.5% eosin (G1100, Solarbio) for 45 s.

*Masson staining:* A Masson trichrome staining kit (G1340, Solarbio) was used. Sections were stained with Weigert’s iron hematoxylin for 5 min, followed by differentiation with an acidic differentiation solution for 5 s and anti-blue treatment with tap water for 3 min. Ponceau fuchsin was then applied for 8 min, followed by a 30-second rinse with a weak acid solution, 1-minute treatment with phosphomolybdic acid, and staining with aniline blue for 2 min.

After staining, the sections were sealed with neutral gum, observed, and photographed under an inverted fluorescence microscope. ImageJ was used to analyze goblet cell counts, villus length, and tissue fibrosis.

### ELISA assay

Diamine oxidase (DAO, SEKR-0122), C-reactive protein (CRP, SEKR-0017), interleukin-6 (IL-6, SEKR-0005), norepinephrine (NE, SEKSM-0019), and tumor necrosis factor-α (TNF-α, SEKR-0009) ELISA kits were purchased from Solarbio (Beijing, China). Lipopolysaccharide (LPS, RF10632) and D-lactic acid (D-LA, RF10851) ELISA kits were obtained from Ruifan Biotechnology Co., Ltd. (Shanghai, China), and the angiotensin II (Ang II, ml058193) ELISA kit was sourced from MLbio (Shanghai, China). Blood samples were collected from the rats in each group, and the levels of DAO, CRP, LPS, D-LA, IL-6, NE, TNF-α, and Ang II were measured according to the instructions provided with each ELISA kit.

### Sympathetic nerve regulation detection

The biological function test system (BL-420S, Yilian Medicine, Shanghai, China) recorded the pulse waveform spectrum of the rat’s tail artery. Electrodes were attached to the muscle layer of the rat. Signals were collected using a biological function signal collector (models EG100 C, UIM100 C) and focused on the low-frequency (LF) range. The LF component of the power spectral density of the pulse wave was then calculated using the TM_WAVE biological signal analysis software. LF is considered a marker of sympathetic nerve regulation.

### Cyclic adenosine monophosphate (cAMP) and cyclic guanosine monophosphate (cGMP) assay

The levels and ratios of cAMP and cGMP in rat plasma were measured according to the protocol of the cAMP and cGMP ELISA kits (Beijing North Institute of Biotechnology, Beijing, China). Rat plasma was centrifuged at 4 ^∘^C and 1000 r/min for 15 min, and the supernatant was collected. Then, 50 µL of supernatant was mixed with 50 µL of Working Solution A, incubated at 37 ^∘^C for 1 h, and washed with washing solution. Afterward, 100 µL of Working Solution B was added and incubated at 37 ^∘^C for 30 min, followed by another wash with washing solution. Finally, 90 µL of substrate solution was added and incubated at 37 ^∘^C for 20 min. The reaction was then terminated by adding 50 µL of termination solution, and the OD value of each well was measured at a wavelength of 450 nm using a microplate reader.

### Ethical statement

This study was approved by Chongqing General Hospital, Chongqing University.

### Statistical analysis

Each experiment was repeated at least three times. Data from measurements that followed a normal distribution are presented as mean ± standard deviation. Statistical analysis and figure generation were performed using Graphpad 9.0. A Student’s *t*-test was used to analyze differences between two groups, while one-way ANOVA was used for comparisons across multiple groups. *P* < 0.05 was considered statistically significant.

## Results

### Exercise can reduce blood pressure and blood lipids in SHR

**Figure 1.**
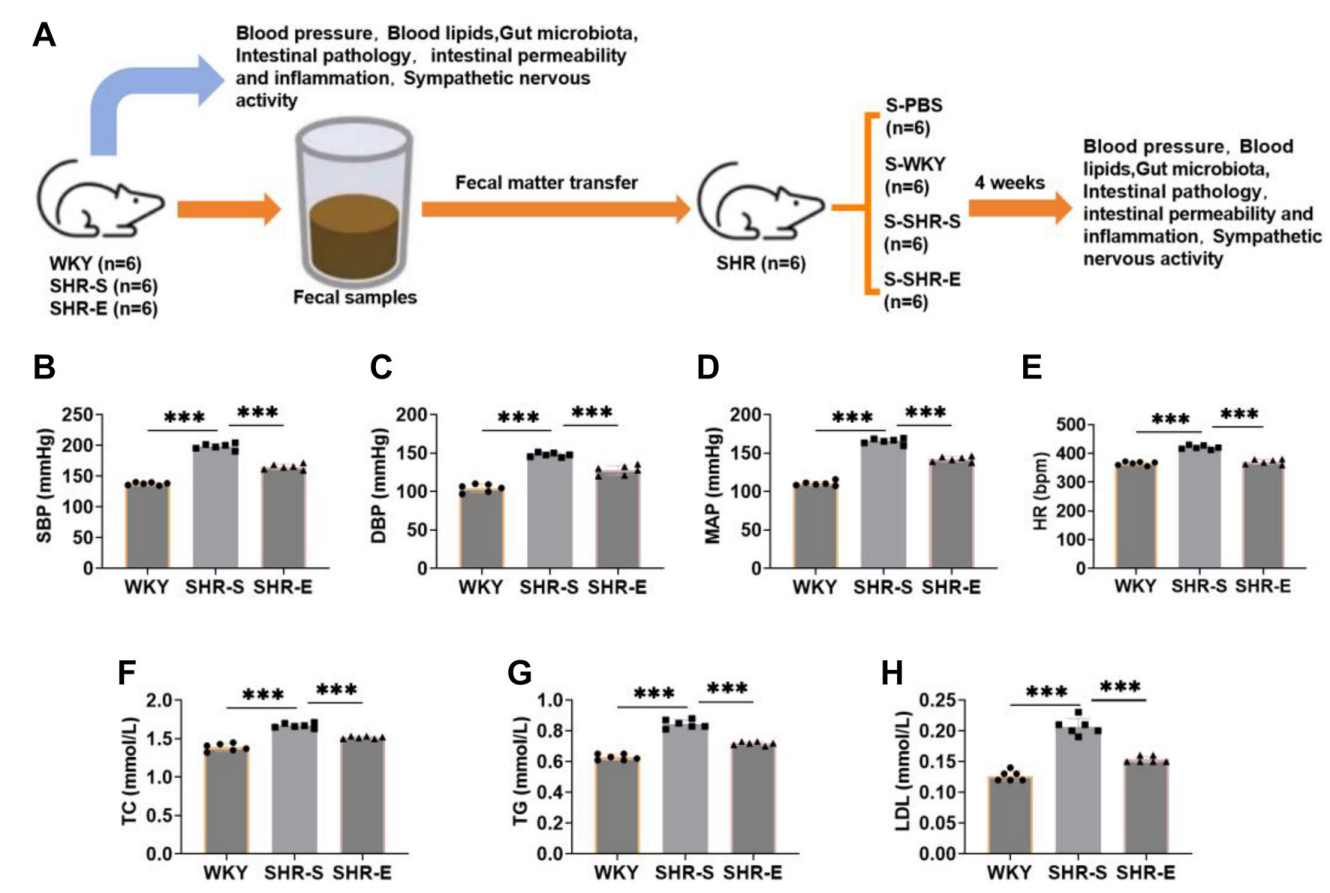
**Exercise can reduce blood pressure and blood lipids in SHR.** (A) Flow chart of experiment. (B–E) SHR were divided into SHR-S group and SHR-E group. The SHR-S group was quiet, and the SHR-E group was treated with a combination of resistance exercise and aerobic exercise for eight weeks. The SBP, DBP, MAP, and HR of the arterial tail of the rats were detected by the blood pressure meter. The blood pressure index of the rats diminished markedly after exercise intervention. (F–H) Automatic biochemical analyzer was used to detect TC, TG, and LDL in serum. The blood lipid index of rats diminished notably after exercise intervention. *n* ═ 6, ***P < 0.001. SBP: Systolic blood pressure; DBP: Diastolic blood pressure; MAP: Mean arterial pressure; HR: Heart rate; TC: Total cholesterol; TG: Triglycerides; LDL: Low-density lipoprotein; WYK: Wistar Kyoto; SHR: Spontaneously hypertensive rats; SHR-E: Exercise-treated SHR; SHR-S: Sedentary SHR.

We investigated the effects of a combination of resistance and aerobic exercise, as well as fecal bacteria transplantation, on spontaneous hypertension in SHR. The experimental workflow is illustrated in Figure 1A. To assess the impact of exercise on blood pressure in SHR, we measured SBP, DBP, MAP, and HR using a blood pressure monitor. Results showed that SBP, DBP, MAP, and HR were significantly elevated in SHR, but these indicators decreased markedly following exercise intervention (Figure 1B–1E), indicating that exercise can effectively lower blood pressure in SHR.

Since hypertension is associated with lipid metabolism disorders [26], we also measured blood lipid levels in SHR. As shown in Figure 1F–1H, exercise significantly reversed the elevated levels of TC, TG, and LDL in the SHR-S group, suggesting that exercise reduces blood lipid levels in SHR. In summary, exercise exerts a therapeutic effect on SHR.

### Exercise can improve the intestinal microflora of SHR

**Figure 2.**
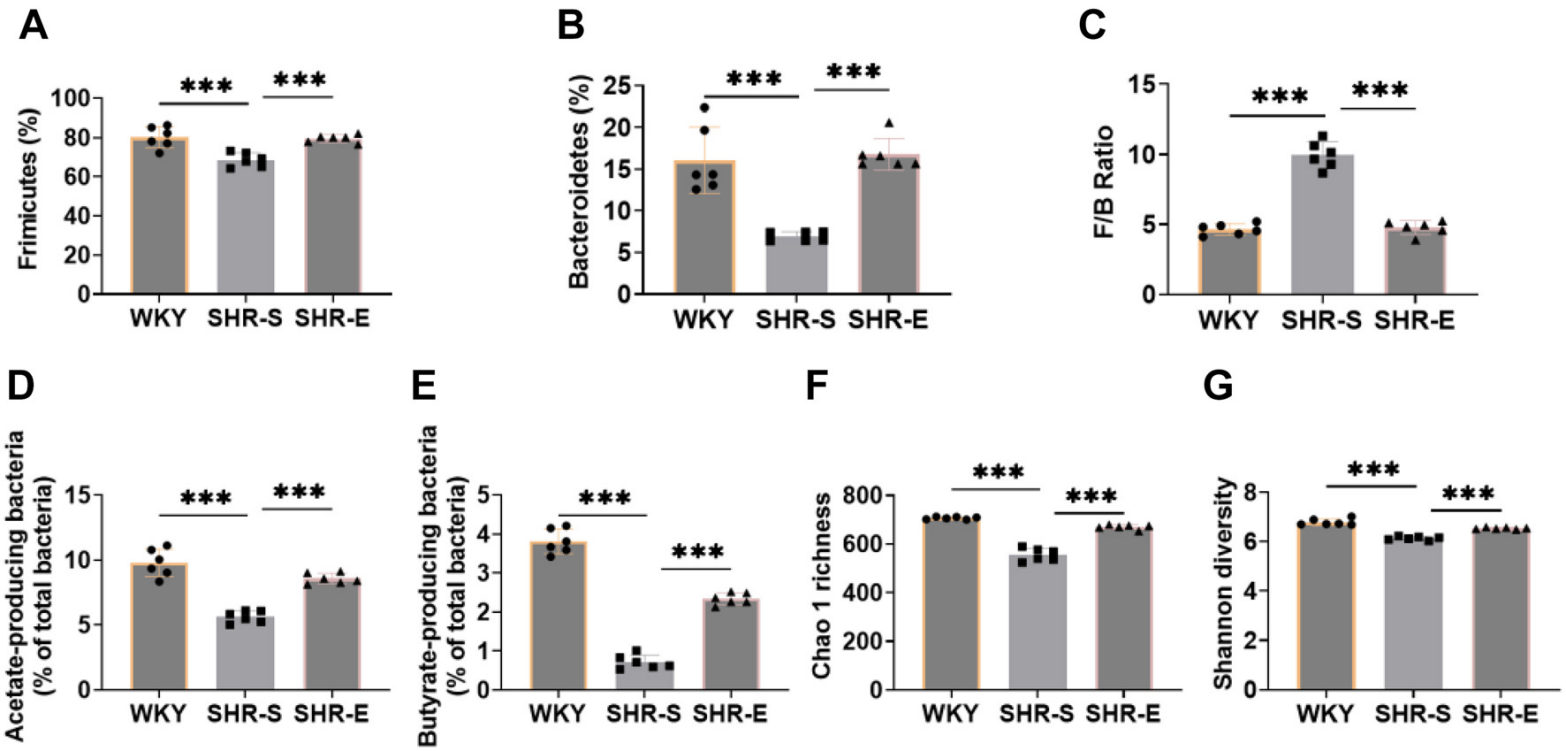
**Exercise can improve the intestinal microflora of SHR.** (A–E) 16S rRNA sequencing analysis of fecal intestinal flora, the number of bacteria producing acetic acid and butyric acid, it can be seen that the number of bacteria increased significantly after exercise intervention; (F and G) Chao 1 richness and Shannon diversity were analyzed by 16S rRNA sequencing. It can be seen that α diversity increased significantly after exercise intervention. *n* ═ 6, ***P < 0.001. F/B: Firmicutes/Bacteroidetes; WYK: Wistar Kyoto; SHR: Spontaneously hypertensive rats; SHR-E: Exercise-treated SHR; SHR-S: Sedentary SHR.

To investigate whether exercise impacts blood pressure and blood lipid levels in SHR through changes in intestinal microbial flora, we performed 16S rRNA sequencing. First, we analyzed the composition of the intestinal flora. Firmicutes and Bacteroidetes were the most prevalent taxa, with their ratio (F/B ratio) serving as a biomarker for the degree of pathology. Hypertension has been linked to an imbalance in intestinal microbial flora, often marked by an increase in the F/B ratio [27]. In comparison to WKY rats, the SHR-S group showed a significant decrease in the abundance of Firmicutes and Bacteroidetes and an elevated F/B ratio (Figure 2A–2C). This imbalance was notably reversed after exercise intervention, suggesting that exercise can ameliorate the intestinal ecological imbalance in SHR rats, potentially contributing to improvements in blood pressure.

We also examined acetic acid- and butyric acid-producing flora as indicators of the metabolic activity of the intestinal microflora. The abundance of these beneficial microbial groups declined significantly in the SHR-S group but increased following exercise intervention, indicating that exercise can enhance the metabolic activity of the flora and improve intestinal function (Figure 2D and 2E).

**Figure S1.**
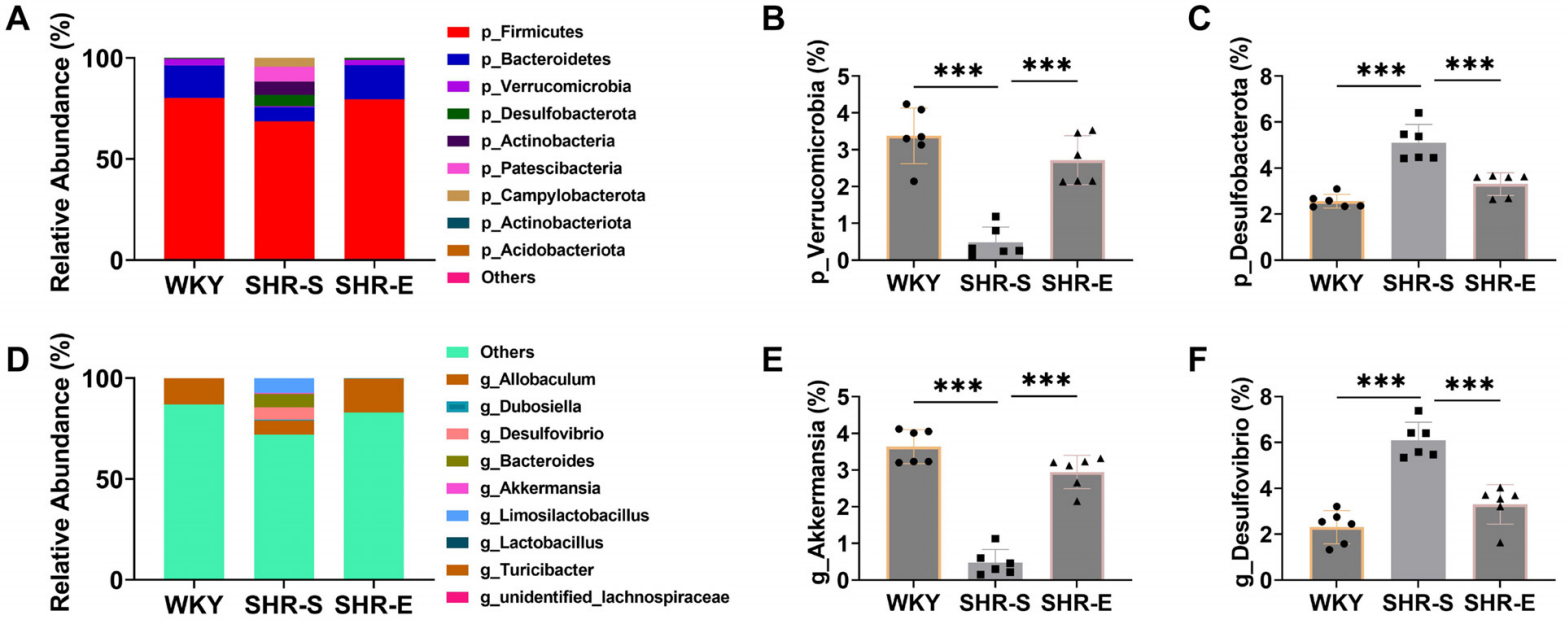
**(A) Summary of the relative abundance of fecal microbiota at the phylum level.** (B and C) At the phylum level, the relative abundance of Verrucomicrobia and Desulfobacterota changed. It can be seen that the relative abundance of Verrucomicrobia increased significantly after exercise intervention, and the relative abundance of Desulfobacterota decreased significantly. (D) Summary of the relative abundance of fecal microbiota at the genus level. (E and F) At the genus level, the relative abundance of Akkermansia and Desulfovibrio changed. It can be seen that the relative abundance of Akkermansia increased significantly and the relative abundance of Desulfovibrio decreased significantly after exercise intervention. WYK: Wistar Kyoto; SHR: Spontaneously hypertensive rats; SHR-E: Exercise-treated SHR; SHR-S: Sedentary SHR.

Additionally, compared with WKY rats, the SHR-S group exhibited a significant reduction in the relative abundance of Verrucomicrobia and Akkermansia, alongside an increase in Desulfobacterota and Desulfovibrio. These changes were also reversed after exercise intervention (Figure S1A--S1F). Finally, α diversity indices were used to assess the richness and diversity of the microbial communities. The Chao 1 richness and Shannon diversity indices were significantly reduced in the SHR-S group (Figure 2F and 2G), but both increased significantly following exercise intervention.

In summary, these findings suggest that exercise can help improve blood pressure and lipid levels in SHR by enhancing the diversity and richness of intestinal microflora, boosting metabolic activity, and promoting the restoration of intestinal microbial balance.

### Exercise can improve intestinal pathology, intestinal permeability, and inflammation in SHR

**Figure 3.**
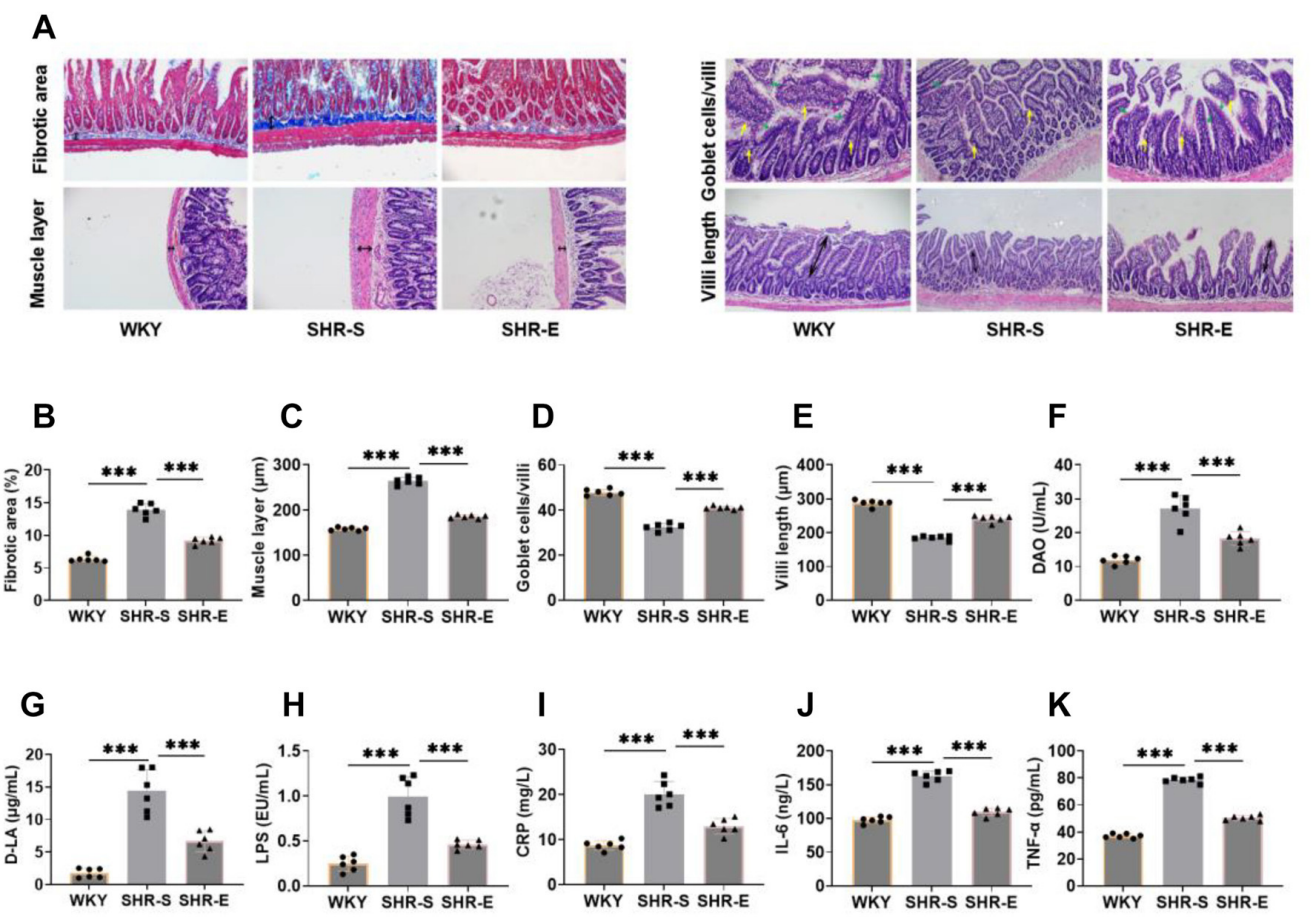
**Exercise can improve intestinal pathology, intestinal permeability and inflammation in SHR.** (A–E) HE and Masson staining were used to test the intestinal pathology of ileum. It can be seen that the thickness of intestinal fibrotic area and muscle layer decreased, and the length of goblet cells and villi increased after exercise intervention (the green arrow represents villi, the yellow arrow represents goblet cells); (F–K) The concentrations of serum DAO, D-LA, LPS, CRP, IL-6 and TNF-α were detected by ELISA kit. It can be seen that the intestinal permeability and inflammation indexes were significantly reduced after exercise intervention. *n* ═ 6, ***P < 0.001. DAO: Diamine oxidase; D-LA: D-lactic acid; LPS: Lipopolysaccharide; CRP: C-reactive protein; IL-6: Interleukin-6; TNF-α: Tumor necrosis factor-α; HE: Hematoxylin–eosin; WYK: Wistar Kyoto; SHR: Spontaneously hypertensive rats; SHR-E: Exercise-treated SHR; SHR-S: Sedentary SHR.

To investigate the impact of exercise on the intestinal tissue of SHR, we first performed HE and Masson staining on the ileum. Compared with WKY rats, the SHR-S group showed increased thickness of intestinal fibrosis and muscle layers, a reduction in goblet cell numbers, and shorter villi (Figure 3A–3E). After exercise, the thickness of the fibrotic area and muscle layer decreased, while goblet cell numbers and villi length increased. These improvements were clearly visible across the measured indicators, suggesting that exercise can alleviate intestinal pathological damage in SHR by preserving the integrity of the intestinal mucosa. Blood pressure regulation is also associated with intestinal epithelial barrier permeability and inflammatory states [28]. Thus, we used DAO and D-LA to assess intestinal permeability, and LPS, CRP, IL-6, and TNF-α to evaluate inflammation. Exercise notably reduced levels of DAO, D-LA, LPS, CRP, IL-6, and TNF-α in the SHR-S group (Figure 3F–3K), indicating that exercise can improve both intestinal permeability and inflammation.

Based on these findings, we speculate that exercise may improve the pathological environment of SHR intestinal tissue and help regulate blood pressure by promoting recovery from intestinal flora imbalance.

### Exercise can reduce the sympathetic nerve activity of SHR

**Figure 4.**
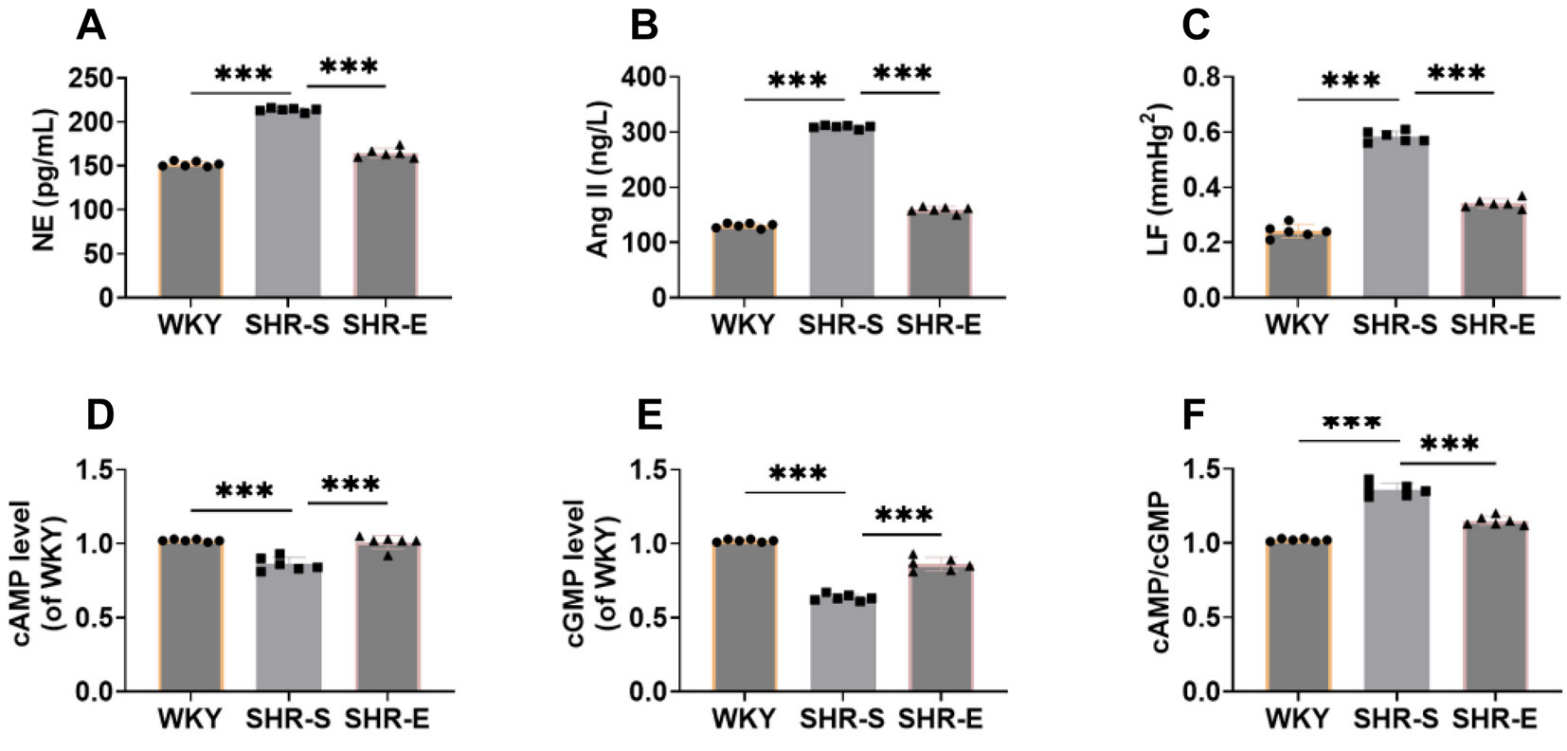
**Exercise can reduce the sympathetic nerve activity of SHR.** (A and B) The levels of plasma NE and Ang II were detected by ELISA kit, which showed that the levels of plasma NE and Ang II were evidently decreased after exercise intervention; (C) The biological function test system analyzes the pulse waveform spectrum of the tail artery of the rat, and calculates the LF. It can be seen that the LF is significantly reduced after exercise intervention; (D–F) ELISA kit was used to detect the levels and ratios of cAMP and cGMP in plasma. The levels of cAMP and cGMP were evidently enhanced and the ratios were significantly decreased after exercise intervention. *n* ═ 6, ***P < 0.001. NE: Norepinephrine; Ang II: Angiotensin II; LF: Low-frequency; cAMP: Cyclic adenosine monophosphate; cGMP: Cyclic guanosine monophosphate; WYK: Wistar Kyoto; SHR: Spontaneously hypertensive rats; SHR-E: Exercise-treated SHR; SHR-S: Sedentary SHR.

Excessive sympathetic nerve activation is a known pathogenic factor in hypertension. NE and Ang II are often used as indicators of sympathetic nerve activity [29]. Compared with the WKY group, levels of NE and Ang II were significantly elevated in the SHR-S group (Figure 4A and 4B) but decreased notably after exercise intervention. Additionally, the strength of the LF components can indicate the stability of the sympathetic nervous system [30]. The LF component in the SHR-S group was markedly increased (Figure 4C). Sympathetic nerve activity is also associated with cAMP and cGMP [31]. In the SHR-S group, cAMP and cGMP levels were significantly decreased, while the cAMP/cGMP ratio was significantly elevated (Figure 4D–4F). After exercise intervention, these indicators were substantially reversed, suggesting that exercise can inhibit sympathetic nerve activity in SHR.

In summary, our findings, along with previous studies, suggest that exercise may help regulate blood pressure by restoring intestinal flora balance in SHR, improving the pathological state of intestinal tissue, and mitigating excessive sympathetic nerve activity. This provides evidence that exercise has a beneficial therapeutic effect on SHR rats.

### SHR-E fecal microbiota transplantation can reduce blood pressure and blood lipids in SHR

**Figure 5.**
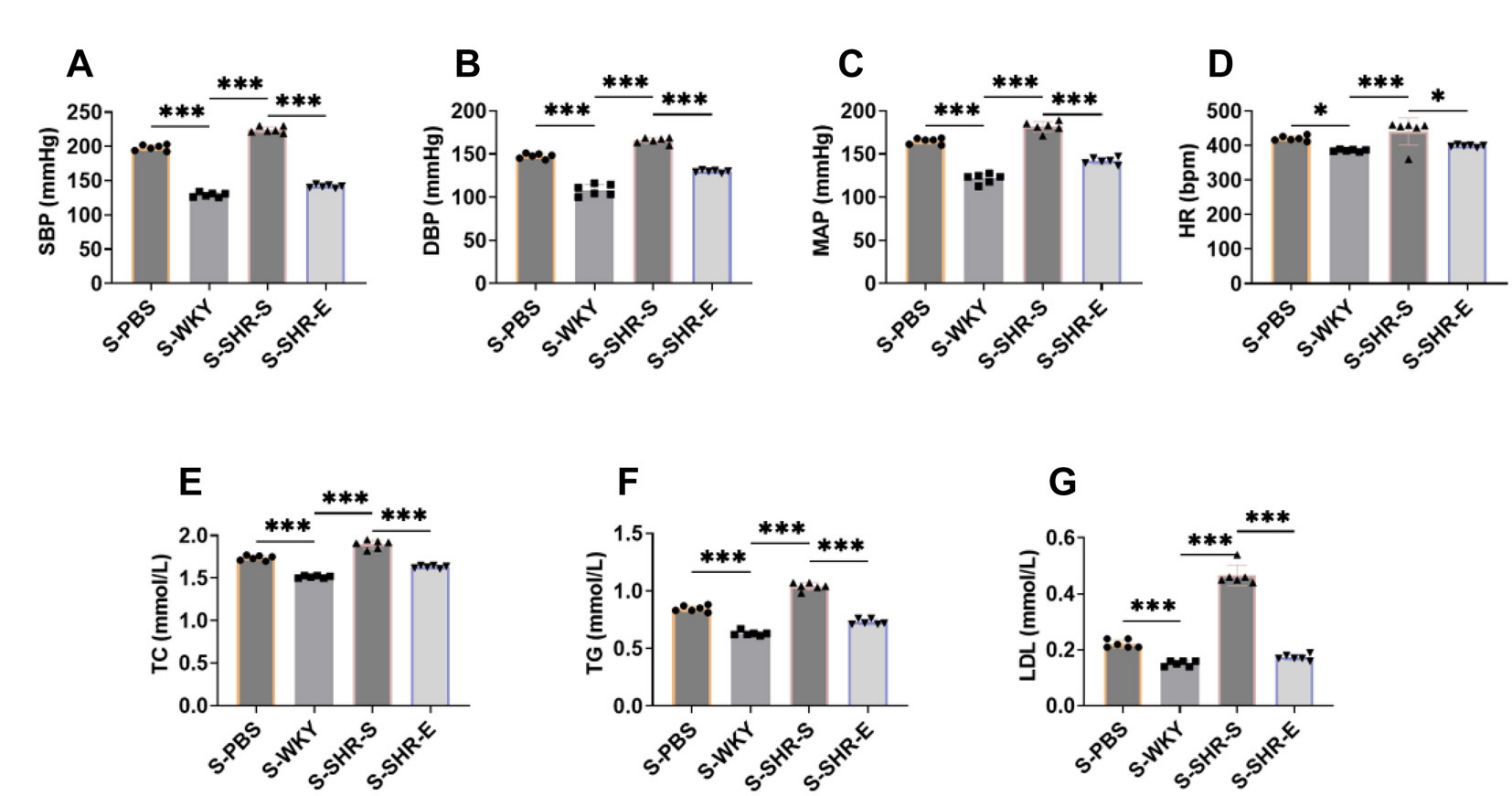
**SHR-E fecal microbiota transplantation can reduce blood pressure and blood lipids in SHR.** (A–D) PBS buffer was used to deplete the intestinal flora of SHR as the blank group. The S-WKY, S-SHR-S, and S-SHR-E groups were enemased with the corresponding fecal bacteria solution once a day, and then exercise intervention was performed. The SBP, DBP, MAP and HR of the arterial tail of the rats were detected by blood pressure meter. The blood pressure index of SHR was significantly decreased after fecal bacteria transplantation. (E–G) The levels of TC, TG and LDL in serum were tested by automatic biochemical analyzer. The blood lipid indexes of SHR were significantly decreased after fecal bacteria transplantation. *n* ═ 6, *P < 0.05, ***P < 0.001. SBP: Systolic blood pressure; DBP: Diastolic blood pressure; MAP: Mean arterial pressure; HR: Heart rate; TC: Total cholesterol; TG: Triglycerides; LDL: Low-density lipoprotein; WYK: Wistar Kyoto; SHR: Spontaneously hypertensive rats; SHR-E: Exercise-treated SHR; SHR-S: Sedentary SHR.

To investigate whether the gut microbiota altered by exercise has therapeutic potential for SHR, we conducted a fecal microbiota transplantation experiment and measured related indicators. The results showed that, compared to the S-WKY group, the blood pressure indicators—SBP, DBP, MAP, and HR (Figure 5A–5D)—and blood lipid indicators—TC, TG, and LDL (Figure 5E–5G)—were significantly elevated in the S-PBS group. These indexes were even higher in the S-SHR-S group, suggesting that fecal transplants from sedentary rats may exacerbate the condition in SHR, supporting the hypothesis that SHR pathology is linked to gut flora imbalance.

In contrast, the blood pressure and lipid indicators in the S-SHR-E group were noticeably reduced compared to those in the S-SHR-S group, indicating that fecal microbiota transplantation from exercised SHR can lower blood pressure and blood lipid levels in SHR. This suggests that the gut microbiota altered by exercise has a beneficial therapeutic effect on SHR.

### The transplantation of SHR-E fecal microbiota can improve the intestinal microbiota, intestinal pathology, intestinal permeability and inflammation of SHR

**Figure 6.**
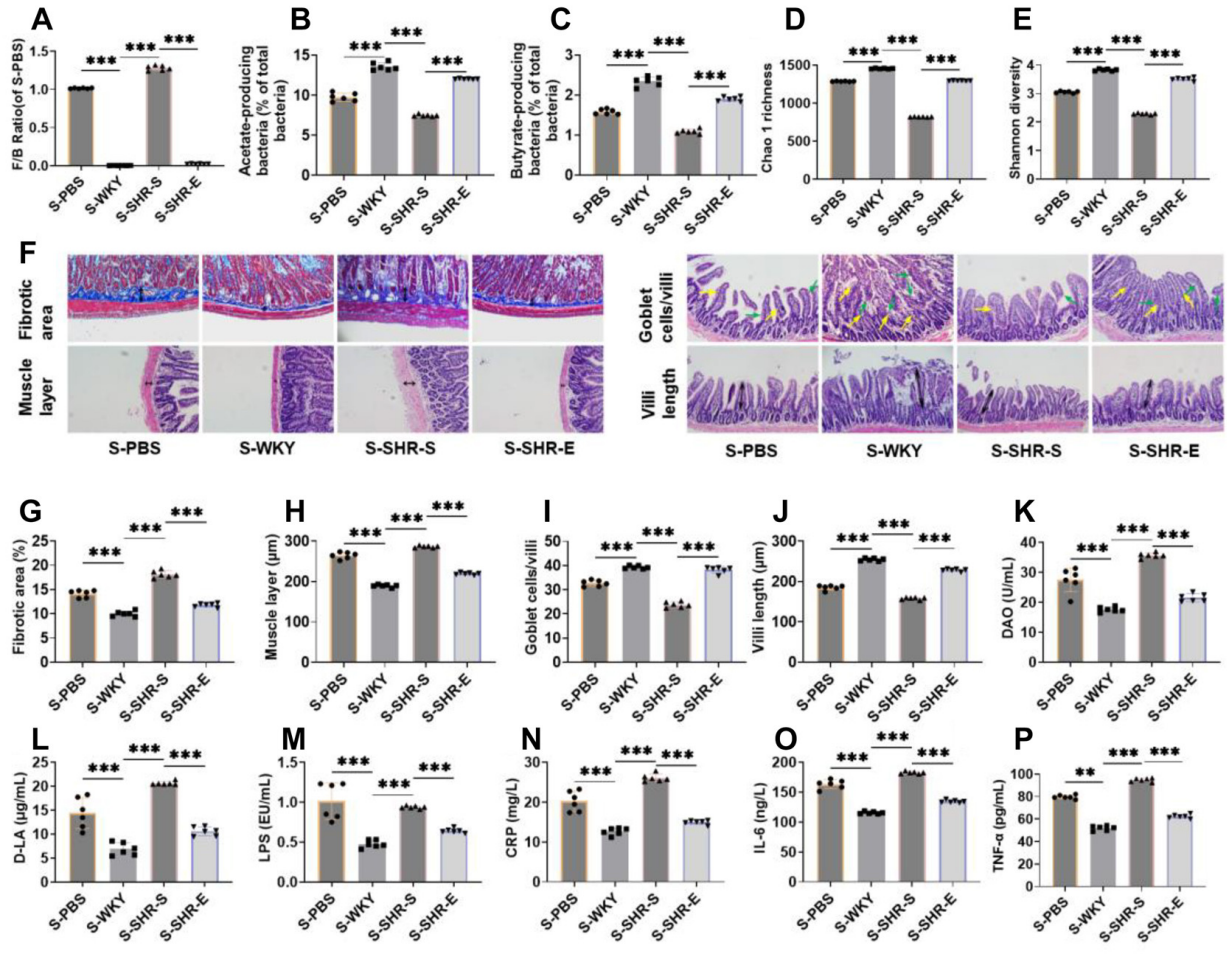
**The transplantation of SHR-E fecal microbiota can improve the intestinal microbiota, intestinal pathology, intestinal permeability and inflammation of SHR.** (A–C) 16S rRNA sequencing was used to analyze the fecal intestinal flora and the number of bacteria producing acetic acid and butyric acid. After fecal bacteria transplantation, the F/B ratio decreased significantly, and the number of bacteria producing acetic acid and butyric acid increased significantly; (D and E) 16S rRNA sequencing analysis showed that Chao 1 richness and Shannon diversity were evidently increased after fecal microbiota transplantation; (F–J) Hematoxylin-eosin and Masson staining were used to test the intestinal pathology of the ileum. After fecal bacteria transplantation, the thickness of the fibrotic area and muscle layer of the ileum of SHR decreased, and the length of goblet cells / villi and villi increased (the green arrow represents villi, the yellow arrow represents goblet cells); (K–P) The concentrations of serum DAO, D-LA, LPS, CRP, IL-6 and TNF-α were detected by ELISA kit. The intestinal permeability and inflammation indexes of SHR were significantly decreased after fecal bacteria transplantation. *n* ═ 6, **P < 0.01, ***P < 0.001. F/B: Firmicutes/Bacteroidetes; DAO: Diamine oxidase; D-LA: D-lactic acid; LPS: Lipopolysaccharide; CRP: C-reactive protein; IL-6: Interleukin-6; TNF-α: Tumor necrosis factor-α; WYK: Wistar Kyoto; SHR: Spontaneously hypertensive rats; SHR-E: Exercise-treated SHR; SHR-S: Sedentary SHR.

**Figure S2.**
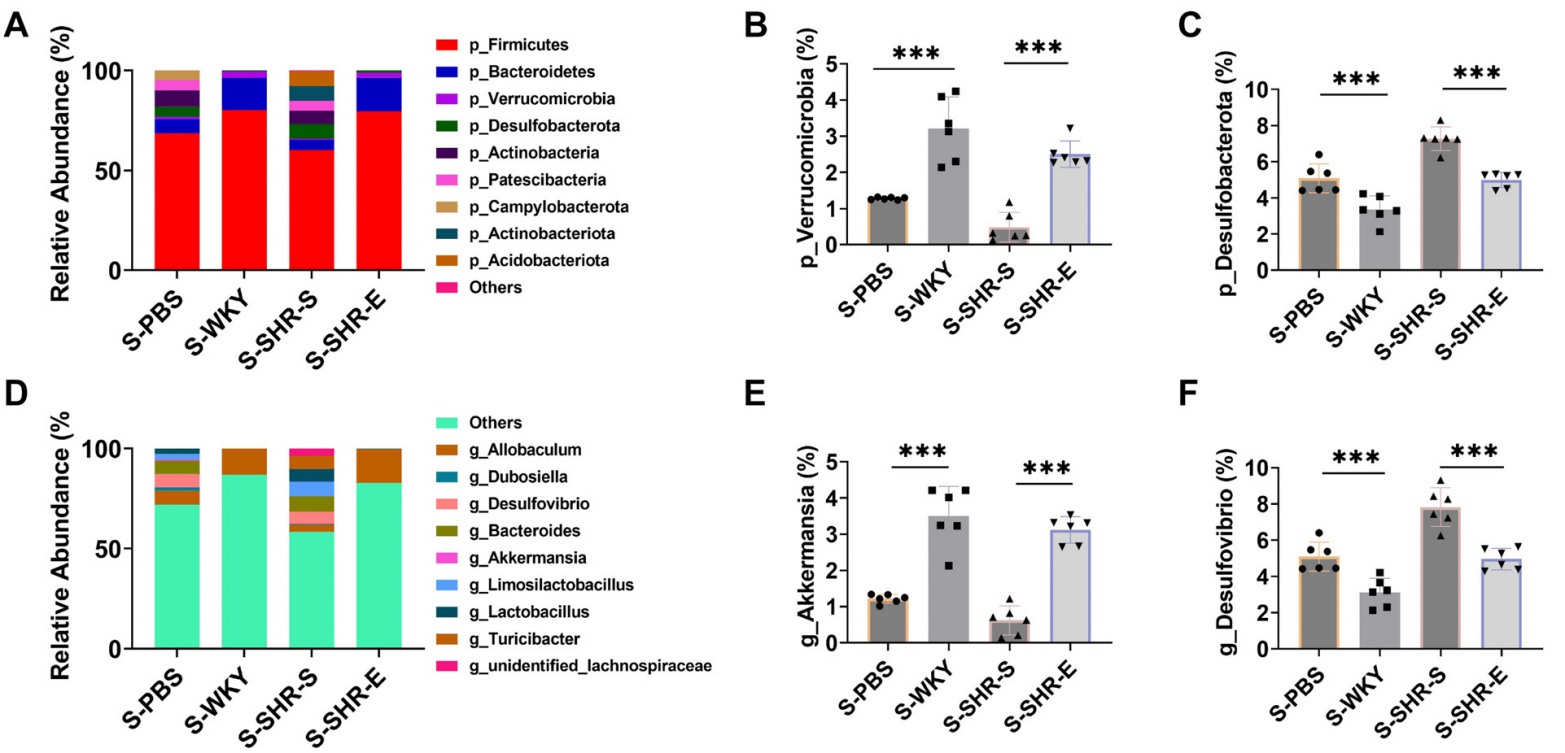
**(A) Summary of the relative abundance of fecal microbiota at the phylum level.** (B and C) At the phylum level, the relative abundance of Verrucomicrobia and Desulfobacterota changed. After fecal bacteria transplantation, the relative abundance of Verrucomicrobia increased significantly, and the relative abundance of Desulfobacterota decreased significantly. (D) Summary of the relative abundance of fecal microbiota at the genus level. (E and F) At the genus level, the relative abundance of Akkermansia and Desulfovibrio changed. After fecal bacteria transplantation, the relative abundance of Akkermansia increased significantly, and the relative abundance of Desulfovibrio decreased significantly. WYK: Wistar Kyoto; SHR: Spontaneously hypertensive rats; SHR-E: Exercise-treated SHR; SHR-S: Sedentary SHR.

To assess the effect of intestinal microflora on SHR intestinal health, we first examined changes in the intestinal microflora of SHR rats. Using 16S rRNA sequencing, we found that, compared with the S-WKY group, the F/B ratio in the S-PBS group was markedly elevated, and the populations of bacteria producing acetic and butyric acid were significantly reduced. Additionally, both Chao 1 richness and Shannon diversity were significantly lower (Figure 6A–6E). The relative abundance of Verrucomicrobia and Akkermansia was significantly decreased, while Desulfobacterota and Desulfovibrio were significantly increased (Figure S2A–S2F). These trends were similarly observed in the S-SHR-S group. However, following the transplantation of SHR-E fecal microbiota, the S-SHR-E group showed a marked reversal of these indices compared to the S-SHR-S group, indicating that SHR-E fecal microbiota transplantation can enhance the diversity and richness of SHR intestinal microbiota, improve its metabolic activity, and support the recovery of a healthier intestinal microbiota profile. HE and Masson staining further demonstrated that, compared with the S-WKY group, the S-PBS and S-SHR-S groups showed increased thickness of intestinal fibrosis and muscle layer, along with reduced lengths of goblet cells and villi and significantly larger lesion areas (Figure 6F–6J). However, after SHR-E fecal microbiota transplantation, these intestinal structure indicators were notably improved in the S-SHR-E group compared to the S-SHR-S group, suggesting that SHR-E fecal microbiota transplantation can restore intestinal mucosal integrity.

Finally, ELISA measurements revealed that, compared with the S-WKY group, the S-PBS and S-SHR-S groups exhibited increased levels of DAO, D-LA, LPS, CRP, IL-6, and TNF-α (Figure 6K–6P). After SHR-E fecal microbiota transplantation, these inflammatory and permeability markers were significantly reduced, indicating that exercise-modulated fecal microbiota transplantation can have a therapeutic effect by improving intestinal permeability and reducing inflammation.

### SHR-E fecal microbial flora transplantation reduces SHR sympathetic nerve activity

**Figure 7.**
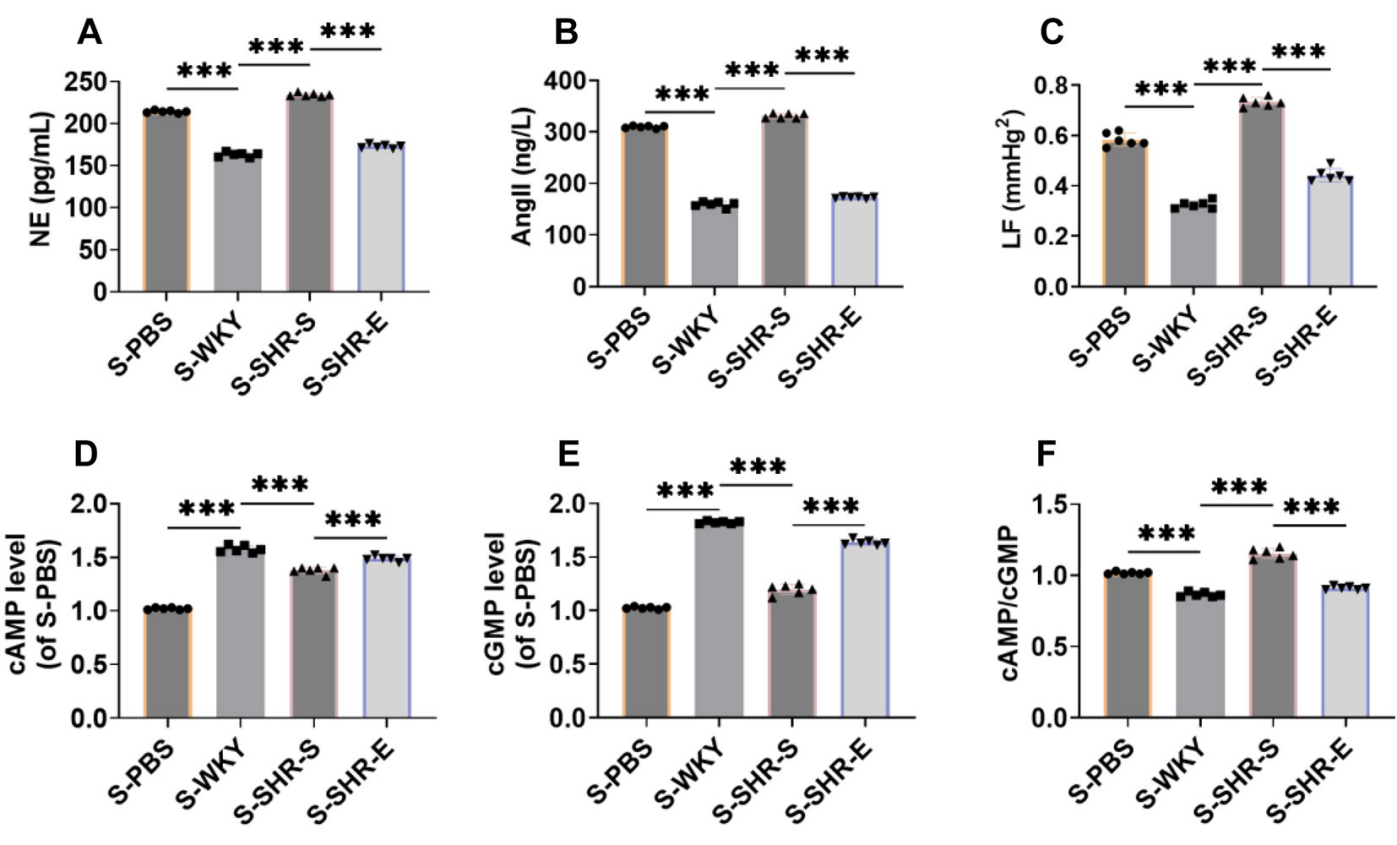
**SHR-E fecal microbial flora transplantation reduces SHR sympathetic nerve activity.** (A and B) The levels of plasma NE and Ang II were tested by ELISA kit. The levels of NE and Ang II were significantly decreased after fecal bacteria transplantation (*P* < 0.001); (C) The pulse waveform spectrum of the rat tail artery was analyzed by the biological function test system, and the IF was calculated. The LF component was significantly reduced after fecal bacteria transplantation; (D–F) The levels and ratios of cAMP and cGMP in plasma were detected by ELISA kit. The levels of cAMP and cGMP were significantly increased, and the ratios were significantly decreased after fecal microbiota transplantation. *n* ═ 6, ***P < 0.001. NE: Norepinephrine; Ang II: Angiotensin II; LF: Low-frequency; cAMP: Cyclic adenosine monophosphate; cGMP: Cyclic guanosine monophosphate; WYK: Wistar Kyoto; SHR: Spontaneously hypertensive rats; SHR-E: Exercise-treated SHR; SHR-S: Sedentary SHR.

We investigated the effect of intestinal microflora on the sympathetic nerve activity in SHR. Using an ELISA kit, we measured NE and Ang II levels and observed a marked increase in the S-SHR-S group (Figure 7A and 7B). LF power was also measured and found to be significantly elevated (Figure 7C). Additionally, levels of cAMP and cGMP, along with their ratio, were assessed using an ELISA kit. We observed a significant decrease in cAMP and cGMP levels, with a corresponding increase in the cAMP/cGMP ratio (Figure 7D–7F). However, following SHR-E fecal microbial flora transplantation, these markers were notably reversed, suggesting that the SHR-E fecal microbial flora could reduce sympathetic nerve activity in SHR.

In conclusion, our study indicates that exercise-derived fecal microbiota can help regulate blood pressure by restoring intestinal flora balance in SHR, improving the pathological state of intestinal tissue, and modulating excessive sympathetic nerve activity. This suggests that the fecal microbiota of SHR-E rats has a positive therapeutic effect on SHR rats.

## Discussion

Hypertension is one of the most common cardiovascular risk factors and a leading cause of death [32]. Its pathology involves multiple interconnected processes, including inflammatory response, lipid imbalance, abnormal activation of the RAAS, and intestinal flora disorders [33–35]. Hypertension is characterized by elevated SBP/DBP [5] and is often accompanied by dyslipidemia [26, 36]. The results of this study show that exercise can reduce SBP, DBP, MAP, and HR in SHR. Additionally, exercise was found to lower serum levels of TC, TG, and LDL, indicating that it can effectively reduce both blood pressure and blood lipids in SHR.

Intestinal homeostasis, essential for overall health, is primarily maintained through interactions between the microbiota and the intestinal epithelial barrier. An imbalance in these systems may contribute to hypertension [37]. Intestinal flora plays a key role in the pathogenesis and treatment of hypertension, as its metabolites can influence blood pressure, risk factors for hypertension, and its associated complications [38–42]. Firmicutes and Bacteroidetes are dominant beneficial bacteria crucial for metabolic health and disease prevention [43]. Their relative abundance, expressed as the Firmicutes/Bacteroidetes (F/B) ratio, can reflect intestinal homeostasis and serve as a biomarker for assessing pathological conditions; thus, an altered F/B ratio is often associated with hypertension [27].

Akkermansia, part of the Verrucomicrobia phylum, is considered a potential probiotic due to its ability to utilize and degrade gastrointestinal mucins, release carbon and nitrogen sources for its own survival, and regulate metabolites, such as SCFAs, LPS, and hydrogen sulfide. Its abundance is influenced by diet and mucin levels and has been present in the human gut microbiota since infancy, increasing into adulthood. Akkermansia abundance is negatively correlated with conditions like obesity, untreated type 2 diabetes, and hypertension [44]. In contrast, Desulfovibrio, a Gram-negative anaerobic bacterium in the gut, is closely associated with LPS production [45, 46]. LPS can permeate the intestinal mucus layer, decompose SCFAs, and produce hydrogen sulfide [47]. It also triggers inflammatory factors like IL-1β, IL-6, and TNF-α, which act on vascular endothelial cells, promote vascular smooth muscle cell proliferation, increase vascular resistance, and ultimately contribute to hypertension [48].

In this study, 16S rRNA sequencing revealed a notable decline in the relative abundance of Firmicutes, Bacteroidetes, Verrucomicrobia, and Akkermansia in SHR, while Desulfobacterota and Desulfovibrio were significantly increased, resulting in an elevated F/B ratio. Following exercise intervention, the F/B ratio was notably reduced, indicating that exercise can help restore intestinal homeostasis by increasing the number of beneficial bacteria. Previous studies have shown that SCFAs can promote endotoxin absorption in the intestine, contributing to blood pressure regulation [49, 50]. Acetate and butyrate make up over half of the SCFAs produced by intestinal microflora, with butyrate primarily produced by Verrucomycaceae, Lachnospiraceae, anaerobic butyric acid bacteria, and anaerobes, while acetate is mainly derived from bifidobacteria and mucin-degrading bacteria [51]. Both acetate and butyrate have been shown to reduce blood pressure [52–54].

Considering the findings that exercise reduces blood pressure in SHR, we hypothesize that exercise may treat hypertension by modulating intestinal microflora, specifically by increasing the abundance of beneficial bacteria and their SCFA production. To validate this hypothesis, we measured the number of bacteria secreting SCFAs. The experimental results demonstrated a significant increase in bacteria producing acetic acid and butyric acid following exercise intervention, suggesting that exercise can help manage hypertension by promoting beneficial bacteria.

In summary, exercise appears to enhance the composition of intestinal microflora in SHR, providing a potential therapeutic pathway for hypertension management.

Previous studies have shown that hypertensive animal models exhibit increased intestinal permeability, decreased expression of tight junction proteins (TJPs), and reduced intestinal villus parameters (both number and length) [55]. This study corroborated these findings. In SHR, we observed increased intestinal fibrosis and muscle layer thickness, a decrease in both goblet cell count and villus length, and significantly elevated serum levels of DAO and D-LA. Following exercise intervention, these changes were notably reversed, indicating that exercise can help maintain intestinal mucosal integrity.

Inflammation is also a critical factor in the pathogenesis and progression of hypertension [56]. LPS can enhance the activity of Ang-II by promoting leukocyte adhesion to the endothelium, activating the RAAS, and thereby causing elevated blood pressure [57]. Additionally, LPS can stimulate the synthesis of pro-inflammatory cytokines, such as tumor necrosis factor-alpha (TNF-α) and IL-6 [58, 59]. Both TNF-α and IL-6 have been shown to directly contribute to the development of hypertension. IL-6 increases the expression of sodium channels in ductal epithelium, enhancing RAAS-mediated water and sodium retention, which raises blood pressure. TNF-α promotes the expression of vascular cell adhesion molecules, encourages microvascular remodeling and sodium retention, reduces NO production, and further contributes to blood pressure elevation [60, 61].

CRP levels are typically low in healthy individuals, but CRP is a major protein involved in inflammation. During acute inflammation or trauma, CRP levels rise significantly, making it a useful marker for assessing the body’s inflammatory response [62]. CRP can also promote the proliferation of vascular endothelial cells and increase arterial wall thickness, contributing to hypertension [63, 64]. In this study, SHR showed significant increases in LPS, CRP, IL-6, and TNF-α levels, all of which decreased significantly after exercise intervention. This suggests that exercise can mitigate hypertension related to inflammation.

In conclusion, exercise can improve intestinal pathology, reduce intestinal permeability, and alleviate inflammation in SHR.

Increased sympathetic nerve activation is recognized as a contributor to hypertension and can occur in the early stages of the condition [65]. Sympathetic nerve activity is often highly elevated in hypertension and can overstimulate the RAAS [66, 67]. Excessive RAAS activation prompts the production of Ang II, which binds to cell membrane receptors, promotes NE release, and triggers various effects, including enhanced vasoconstriction and vascular wall hypertrophy, both of which lead to elevated blood pressure [68]. Additionally, NE can bind to adrenergic receptors and activate Gi protein, which inhibits intracellular cAMP production [69]. Sympathetic activation also releases neuropeptide Y (NPY) and other neurotransmitters through nerve terminals. NPY, on one hand, can activate Gi protein to reduce cAMP levels, and on the other, it can bind to the Y2 receptor to release NO and stimulate cGMP production in cells [31]. As blood pressure becomes more abnormal, the imbalance between vasodilation and vasoconstriction intensifies. Both cAMP and cGMP, important indicators of ion channel activity in cell membranes, play significant roles in vasodilation [70, 71]. The autonomic nervous system—including the sympathetic, parasympathetic, and enteric nervous systems—is essential for maintaining homeostasis. The sympathetic nervous system, specifically, is critical for blood pressure regulation, with the strength of the LF component reflecting nervous system stability [30, 72]. In this study, the levels of NE, Ang II, LF, cAMP, and cGMP, along with their ratios, were significantly elevated in SHR and significantly decreased after exercise intervention. This indicates that exercise can reduce sympathetic activity in SHR.

In recent years, evidence from bacterial genome analysis and fecal microbiota transplantation studies has supported the role of intestinal flora in regulating blood pressure [23]. Fecal microbiota transplantation, particularly using “washed” microbiota, has been shown to effectively reduce blood pressure [73]. In this study, fecal bacteria from exercise-treated SHRs (SHR-E) and sedentary SHRs (SHR-S) were transplanted into other SHRs. Results showed a significant reduction in both blood pressure and blood lipids in the SHR-E group compared to the control. The F/B ratio in the intestinal flora decreased markedly, and the abundance and diversity of acetic acid- and butyric acid-producing bacteria increased significantly, suggesting that fecal microbiota transplantation helps restore the intestinal microflora of SHRs and maintain intestinal homeostasis.

Furthermore, intestinal fibrosis and muscle layer thickness decreased, while the number of goblet cells/, villus length, and goblet cell/villi ratio increased significantly. Levels of DAO, D-LA, LPS, CRP, IL-6, and TNF-α also dropped, indicating improved intestinal mucosal integrity and reduced permeability and inflammation in the SHRs receiving SHR-E fecal microbiota. Additionally, markers related to sympathetic nervous activity, such as NE, Ang II, LF components, cAMP, and cGMP, were significantly reduced, suggesting that SHR-E fecal microbiota transplantation can lower sympathetic nerve activity in SHRs.

In conclusion, fecal microbiota transplantation from exercise-treated SHRs can improve spontaneous hypertension in rats. Combined with previous findings that exercise can alleviate hypertension, this suggests that exercise may help reduce blood pressure partly by remodeling the intestinal flora in SHRs. Similar results have been observed in human studies: germ-free mice receiving fecal transplants from hypertensive patients developed a gut microbiota resembling that of the donor within eight weeks, along with increased systolic and SBP/DBP, demonstrating a direct effect of intestinal microorganisms on host blood pressure[74].

In summary, fecal microbiota transplantation from hypertensive donors, whether human or animal, can elevate blood pressure in normotensive recipients [74, 75]. However, most current research relies on animal models like mice and rats. Differences between human and animal gut microbiota may limit the applicability of these findings to humans, so further studies using models closer to humans—such as non-human primates or humanized mice—are needed. Ultimately, clinical trials are required to screen safe and effective microbial strains based on hypertension severity, variability, and potential toxicity to achieve effective treatment outcomes for hypertension.

## Conclusion

This paper reveals the mechanism by which exercise improves spontaneous hypertension in rats. Exercise can positively impact blood pressure, blood lipids, gut flora, intestinal pathology, intestinal permeability, inflammation, and sympathetic nerve activity in SHR. Its mechanism is linked to changes in intestinal microflora. This study provides a valuable reference for targeted hypertension treatments. Future research could continue investigating how exercise regulates blood pressure via specific microbial strains, such as Akkermansia and Desulfovibrio. However, most current studies on gut microbiota and hypertension focus on correlation rather than causation, and the specific pathways and targets involved remain largely unexplored. Additionally, most mechanistic insights are derived from animal and cell models, with the potential molecular mechanisms still not fully clarified. Therefore, future research could leverage multi-omics approaches and predictive modeling tools to analyze the structure and function of intestinal microorganisms, accurately classify gut flora, and integrate basic and clinical research. This comprehensive approach could deepen understanding of their roles and mechanisms, ultimately providing a foundation for targeted hypertension therapies.

## Supplemental data

**Highlights:**

1. Exercise can reduce blood pressure and blood lipids in spontaneously hypertensive rats (SHR).
2. Exercise can improve the intestinal microflora and intestinal histopathology of SHR.
3. Exercise can reduce the sympathetic nerve activity of SHR.
4. Intestinal microflora in SHR exercise group (SHR-E) can reduce blood pressure, blood lipid and sympathetic nerve activity in SHR.
5. The intestinal microflora of SHR-E rats can improve the intestinal microflora and intestinal histopathology of SHR.

**Figure S3.**
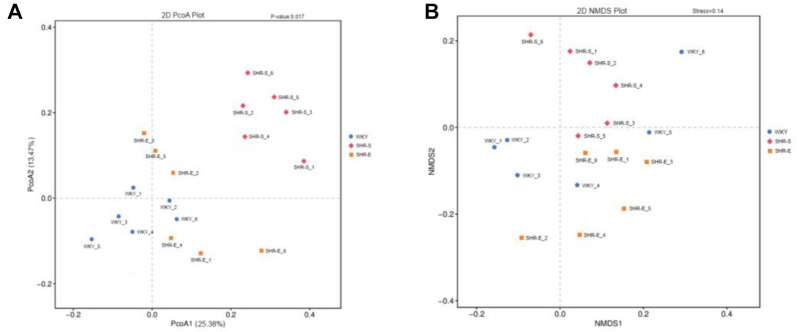
**(A and B) Analysis of β diversity of intestinal flora in rats after exercise.** WYK: Wistar Kyoto; SHR: Spontaneously hypertensive rats.

**Figure S4.**
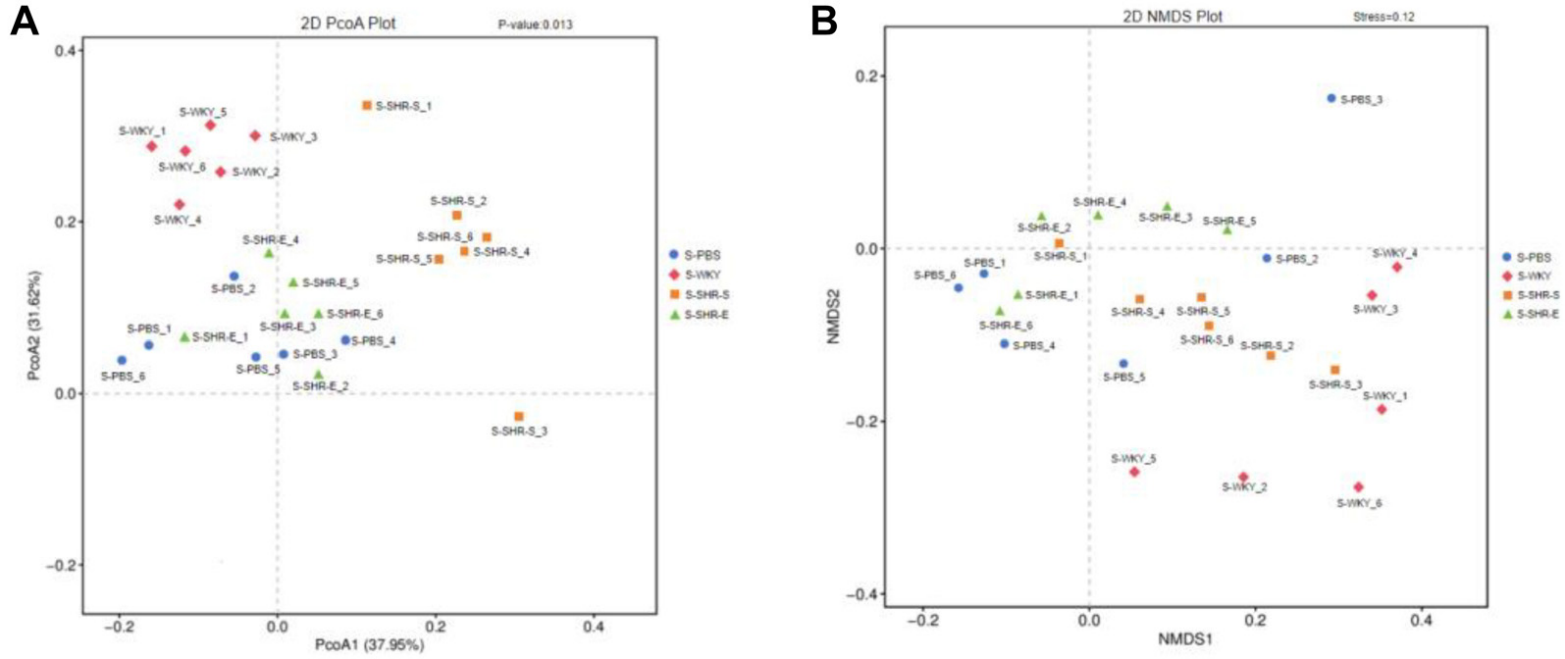
**(A and B) Analysis of β diversity of intestinal flora in rats after fecal bacteria transplantation.** WYK: Wistar Kyoto; SHR: Spontaneously hypertensive rats.

## Data Availability

The data that support the findings of this study are available from the corresponding author, upon reasonable request.
